# Climate change and natural disasters: global challenges for mental health, resilience, and recovery

**DOI:** 10.1192/j.eurpsy.2025.2004

**Published:** 2025-08-26

**Authors:** F. Kraxner, S. Tanyeri Kayahan

**Affiliations:** 1 Psychiatry, Hospital Affoltern am Albis, Zürich, Switzerland; 2 Psychiatry, Republic of Türkiye Ministry of Health Yalvac State Hospital, Isparta, Türkiye

## Abstract

**Introduction:**

Climate change is the biggest threat of the 21^st^ century and poses a significant risk to mental well-being by aggravating social injustice, by which children and adolescents are particularly affected. To avert the scenarios predicted by scientists, it is not only necessary for politicians to act quickly and thoroughly but also to rethink our self-understanding as those responsible for planetary health and to face this crisis with a fundamental rearrangement of priorities.

**Objectives:**

Highlighting and summarizing current knowledge on climate change and its effects on mental health were aimed.

**Methods:**

To emphasize the global challenges related to mental health, facts and figures of climate change and the actual status quo of climate-related mental health were gathered through an online review of existing resources.

**Results:**

According to the Glasgow Climate Pact of the United Nations Framework Convention on Climate Change, the world is currently heading for a global warming of 2.7°C, accepting the destruction of habitats and ecosystems. The warnings regarding climate change from the scientific community are becoming increasingly clear. The Lancet Countdown, a review by scientists from 43 leading institutes around the world, shows visible impacts of climate change on human health. The Intergovernmental Panel on Climate Change report confirms a global increase in climate change-associated morbidity and mortality in 2023, as well as the impact on mental health. The climate crisis threatens mental health ubiquitously. In this context, new terms as “eco-anxiety”, “eco-paralysis”, and “solastalgia” are already being used. Eco-anxiety describes the fear of directly experiencing climate change. While this fear results in a change towards environmentally friendly behavior in some people, others fall into eco-paralysis. Knowledge about climate change can lead to paralysing fear and denial. Trauma from experiencing extreme weather events or forced relocation also trigger psychological distress in young people. In addition to eco-anxiety, the term solastalgia is increasingly used to describe the existential pain caused by experiencing irreversible climate-related changes in the environment. Surveys show that anxiety about the future has increased among young adults in recent years. The interconnections between climate change and mental health also include stress reactions and emotional suffering, strained social relationships, helplessness, grief, and increased risk of suicidal behavior.

**Conclusions:**

Considering many health care professionals do not yet carry out any corresponding preventive measures regarding climate change and its effects on mental health, the self-image of physicians must be rethought and sharpened as communicators and ambassadors. To address this global topic, it is urgent to strengthen research, training, policies, advocacy, and data collection.

**Disclosure of Interest:**

None Declared

